# Long-term follow-up of changes in ocular biometric parameters in orthokeratology lens wearers with relatively large-scale axial length reduction

**DOI:** 10.1186/s40662-022-00324-z

**Published:** 2023-02-02

**Authors:** Tao Tang, Xuewei Li, Sitong Chen, Qiong Xu, Heng Zhao, Kai Wang, Yan Li, Mingwei Zhao

**Affiliations:** 1 Institute of Medical Technology, Peking University Health Science Center, Beijing, China; 2 Department of Ophthalmology and Clinical Centre of Optometry, Peking University People’s Hospital, Beijing, China; 3 College of Optometry, Peking University Health Science Center, Beijing, China; 4 Eye Disease and Optometry Institute, Peking University People’s Hospital, Beijing, China; 5 Beijing Key Laboratory of the Diagnosis and Therapy of Retinal and Choroid Diseases, Beijing, China

**Keywords:** Myopia, Contact lens, Orthokeratology, Axial length, Crystalline lens thickness

## Abstract

**Background:**

To investigate ocular biological characteristics for myopic children with axial length (AL) reduction during orthokeratology (Ortho-K) treatment and provide clinical clues for better myopia control effects.

**Methods:**

Changes in ocular parameters and treatment zone (TZ) in 75 subjects who completed one-year Ortho-K treatment were retrospectively reviewed. The subjects were divided into two groups according to one-year AL change: the AL reduction group (n = 37) and the AL elongation group (n = 38). Univariate and multivariate regression analyses were performed to determine the association between TZ, ocular parameters, and AL change.

**Results:**

There was no significant difference in baseline between the two groups (all *P* > 0.05). After one year of Ortho-K treatment, compared with those in the AL elongation group, children in the AL reduction group had a decreased anterior chamber depth (ACD) (*P* < 0.001), thickened crystalline lens thickness (CLT) (*P* = 0.002), thinned vitreous chamber depth (VCD) (*P* < 0.001) and smaller TZ (*P* = 0.03), but no difference in central corneal thickness (CCT) and pupil diameter (PD). In the multivariable analyses, AL reduction was negatively associated with baseline age (beta: − 0.048; 95% CI: − 0.083 to − 0.013; *P* = 0.009) and positively associated with the TZ (beta: 0.024; 95% CI: 0.009 to 0.040; *P* = 0.003).

**Conclusions:**

In AL reduction eyes, thickened CLT, decreased ACD and thinned VCD were observed during Ortho-K treatment, which could be suggested as indicators for better myopia control effects in the clinic. Older baseline age and smaller TZ wearing Ortho-K were also associated with AL change. Thickened CLT may be a result of compensation for AL-reduction eyes.

## Background

Myopia is an ocular disorder characterized by significant axial length (AL) elongation of the eyeball. Myopic progression in children has become a serious public concern, particularly in East Asia [1–3]. According to population-based epidemiological research, the prevalence of myopia can reach approximately 69% in children aged 15 years old and 80% in children aged 18 years old [4]. To reduce the risk of myopia-related pathological disorders, such as glaucoma, cataract and macular degeneration [5–8], many potential pharmaceutical and optical treatments have been implemented to slow myopia progression in children [9].

Orthokeratology (Ortho-K) lenses are a special type of rigid contact lenses that correct low to moderate myopic refractive error during sleep; they have become increasingly popular for controlling myopia. Statistics estimate that there are more than 1.5 million Ortho-K lens wearers in China [10]. Previous studies related to myopia control using Ortho-K have examined how Ortho-K lenses control the rate of myopia progression. Ortho-K has been reported to effectively slow axial elongation by 43% to 63% [11–13]. This effect might be related to an increase in peripheral myopic defocus induced by corneal shape changes after Ortho-K lens wear [14]. However, there is significant individual variability in the initial myopic refractive error and treatment zone (TZ) which can be affected by age [15–17]. Although Ortho-K is an effective method to control myopia progression, AL still elongates in varying age groups. In the Cho et al.’s study, the average axial elongation was 0.36 ± 0.24 mm in the Ortho-K group over two years, with a slower increase in AL by 43% than that in the control group [11]. In multiple clinical studies related to Ortho-K treatment, ocular AL reduction was rarely observed.

AL change is a crucial parameter in evaluating myopic progression. The most used noncontact AL measurement instrument in clinical practice, such as the IOLMaster, utilize infrared interferometry to measure the distance from the top of the anterior surface of the cornea to the retinal pigment epithelium (RPE). Therefore, changes in the corneal epithelium and choroidal thickness under the RPE will affect changes in the AL [18, 19]. A reduction in AL greater than 0.1 mm over a one-year period in myopic children who wore Ortho-K lenses was observed, which was clearly beyond the range of choroidal thickness variation and thinning of central corneal thickness. In addition, although the reduction in AL can be partly explained by reduced corneal epithelium [20] and increased choroidal thicknesses [21], it was reported in optometry clinics that AL can be reduced by more than 0.1 mm. Thus, this AL reduction cannot be explained only by changes in the corneal epithelium and choroidal thicknesses alone after Ortho-K wear, suggesting that further studies concerning components of AL should be carried out. The analysis of these phenomena may be able to provide clinicians with new perspectives and clinical experience on how to control AL elongation and progression of myopia better in children and adolescents using the Ortho-K lens. The objective of this study was to explore the characteristics of AL component changes in cases of AL reduction over a one-year period in myopic children under Ortho-K treatment.

## Methods

### Study population

**Fig. 1:**
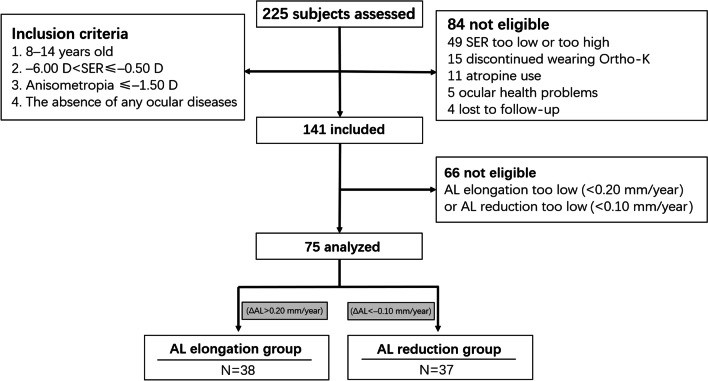
Flow chart of the study. SER, spherical equivalent refraction error; AL, axial length; ΔAL, change in AL (AL at the one-year visit − AL at baseline); Ortho-K, orthokeratology. D, diopters

This was a retrospective study. All data from the examination results were extracted from the device readouts and medical records of children who visited Peking University People’s Hospital optometry center for vision correction with Ortho-K from January 2019 to December 2020. Everyone was assigned a unique identification number. Only parameters of right eyes were collected as individual sample data because of the high correlation between both eyes. The subjects were selected according to the inclusion criteria and excluded if they conformed to one of the situations noted in the exclusion criteria (Fig. 1). The purpose and procedure of this study were explained in detail to the parents or legal guardians of the children, and they provided written informed consent for data storage and data usage for clinical/research purpose before the study. This study was approved by the institutional research ethics committee of Peking University People’s Hospital (2021PHB322-001) and adhered to the tenets of the Declaration of Helsinki.

In total, 75 children (75 eyes) aged 8 to 14 years with low to moderate myopia (−0.50 to −5.00 D), refractive astigmatism no greater than −1.50 D, a spherical equivalent refractive error (SER) of not less than −6.00 D in both eyes, and low anisometropia (≤ 1.50 D) were included. Children who failed to wear their Ortho-K lenses at least three times, had a history of atropine use or any other ocular health problems, or lost to follow-up were excluded from the study.

The subjects were divided into two groups for analysis of axial change. In previous studies, the wearing of Ortho-K lenses will cause approximately 40 μm thickening [19], 24 h rhythm will cause approximately 20 μm choroidal thickness change [22], and corneal thickness will decrease by 10 to 20 μm [19]. To rule out the influence of these factors on axial elongation, we defined the AL reduction group as the group wearing Ortho-K lenses resulting in a shortening of 0.1 mm AL within one year. The increased AL of patients wearing Ortho-K lenses is approximately 0.2 mm per year [23], so the group with an axial elongation of more than 0.2 mm per year is defined as the AL elongation group.

### Ocular examinations

The subjects first watched television at a distance of 5 m for 20 min, then refraction and ocular biometry examinations were performed in all subjects. First, measurements of ocular biometric parameters were taken with an optical biometer. AL, central corneal thickness (CCT), anterior chamber depth (ACD), and crystalline lens thickness (CLT) were measured with noncontact partial-coherence laser interferometry (IOLMaster 500; Carl Zeiss Meditec, Oberkochen, Germany). Vitreous chamber depth (VCD) was calculated by subtracting the CCT, ACD and LT from the AL. A corneal topography system (the Sirius, Italy) was used to obtain the mean keratology reading (Kmean), pupil diameter (PD) and horizontal visible iris diameter (HVID). Five measurements were taken for each AL measurement, and only the repeats with intrasession differences less than 0.02 mm were averaged and recorded. All measurements were performed under the same ambient light and by the same blinded examiner. The ocular parameters are presented as mean ± standard deviation (SD).

After the biometry measurements, all subjects underwent noncycloplegic refraction examination with an autorefractor (model RK-F1; Canon, Tokyo, Japan). This instrument generated five reliable readings of refraction in both eyes; the median reading was used for analysis. Cycloplegic refraction was performed in each subject after the administration of 0.5% compound tropicamide eye drops (Santen Pharmaceutical Co., Ltd., Japan; 0.5% tropicamide combined with 0.5% phenylephrine). Three drops of 0.5% compound tropicamide eye drops were administered at least 25 min before refractive error measurement. Cycloplegia was considered complete if the pupil dilated to 6 mm or more and there was no pupillary reflex. The average of three measurements automatically obtained by the autorefractor was used for analyses.

### Procedure

All Ortho-K subjects were fitted with the same spherical 4-zone lenses (Euclid, Euclid Systems Corporation, Herndon, Virginia, USA), which have a spherical base curve (BC, standard optic zone diameter is 6.2 mm) made of a gas-permeable material. A certified ophthalmic technician performed lens fitting according to the manufacturer’s instructions based on corneal topography, noncycloplegic manifest refraction, and the horizontal visible iris diameter. The subjects wore a trial lens, and the final best-fitting lens was determined by good dynamic fluorescence fitting. Subsequently, the Ortho-K lenses were dispensed to the children. The children were advised to wear the lenses for at least eight consecutive hours every night. Myopic progression was estimated based on the change in the AL in the two groups. AL measurements were obtained using the same IOLMaster instrument each time by the same blinded examiner.

Before the subjects in the two groups started wearing the Ortho-K lenses, their AL, best corrected visual acuity (BCVA), noncycloplegic refraction, cycloplegic refraction, and corneal topography were assessed. AL was used to estimate myopic progression. Subjects were examined at 1, 7, and 30 days and 6 and 12 months after they started wearing the Ortho-K lenses. The examinations were performed 3 h after removing the lenses and included measurements of the subjects’ VA, AL, CCT, ACD, LT, PD, VCD and corneal topography as well as fluorescein staining of the corneal epithelium.

### Definitions

**Fig. 2:**
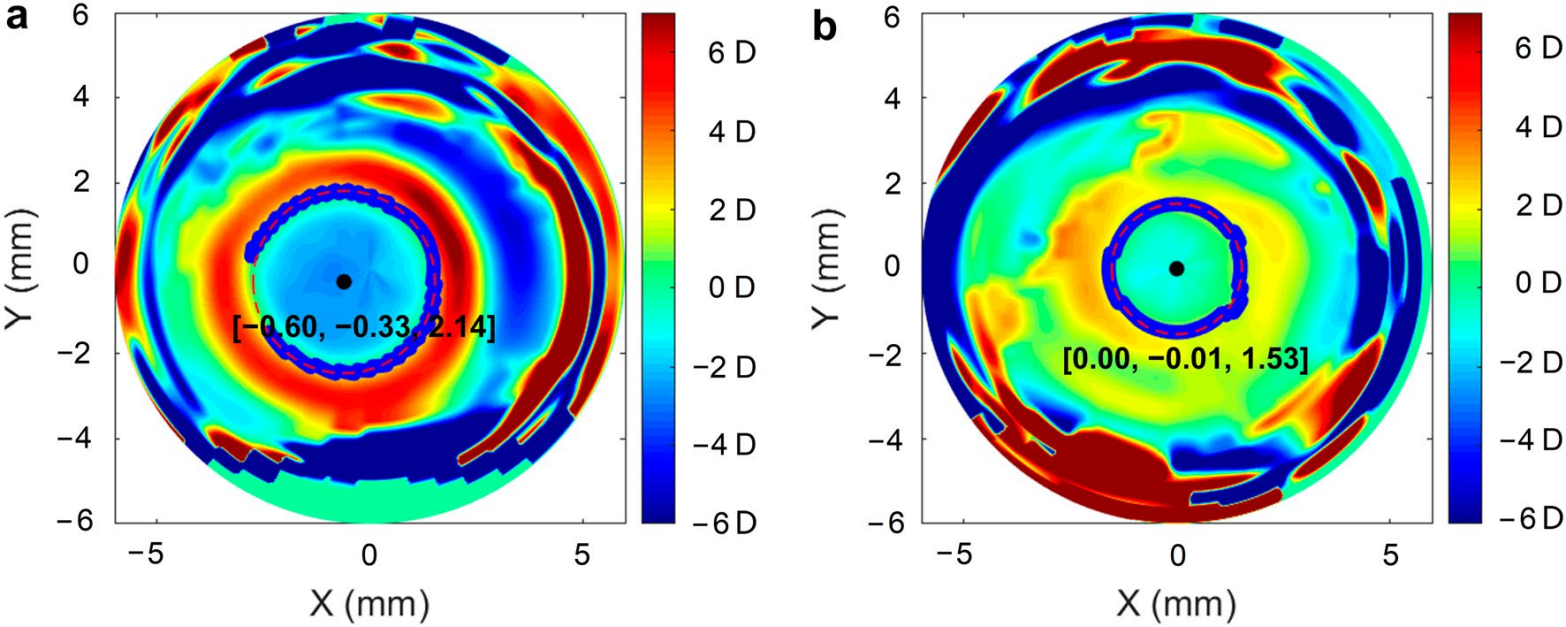
Corneal topography difference map after Ortho-K lens wear for one year. **a** One of the myopic eyes from the AL elongation group. **b** One of the myopic eyes from the AL reduction group. The center coordinates of the treatment zone area are [− 0.60, − 0.33] and [0, − 0.01], respectively, and the radii of the treatment zone area are 2.14 mm and 1.53 mm, respectively

Refraction was defined as spherical equivalent refraction (SER; SER = spherical power + cylinder power/2). The mean K reading [Kmean = (flat K reading + steep K reading)/2] was the average of the steepest and flattest meridians. The corneal radius of curvature (CR) was converted from the Kmean data using the formula CR = 0.3375/Kmean (D) × 1000. During Ortho-K treatment, the degree of myopia is reduced by flattening the central cornea. This central flattened zone is referred to as the TZ [24, 25]. To calculate the TZ area (πr^2^), the CSV files of the corneal sagittal height data were exported from the Sirius system at baseline and after one year of Ortho-K treatment. A customized specific MATLAB program was used to calculate the TZ area as outlined in a previous study [26]. A total of 256 points on the TZ border, defined as the points of transition from negative to positive values, were automatically extracted from the tangential difference map. The best fitting radius of “ring” for these points was calculated by the least squares method. The TZ area can be calculated as πr^2^. Tangential difference maps centered in the geometric cornea; the decentration of the treatment zone can also be deduced from this ring (Fig. 2).

### Statistical analysis

PASS 11.0 (NCSS Statistical Software, Kaysville, UT, USA) was used for statistical sample size. According to the design of the comparison in the difference between the two groups in the pre-experiment, a sample size of 23 in each group was determined to be required for a power of 0.90 and an a-value of 0.05. The data obtained were analyzed using the SPSS statistical software package (Version 22.0, IBM Corp., US). For eye-specific measurements, only data from the right eye of each participant were included for analysis. Continuous data are presented as means ± SD. The TZ was calculated using the MATLAB 2018a software package (The MathWorks, Inc., USA). The difference in the male/female (M/F) ratio between the groups was assessed using the Chi-squared test. Paired t-tests were used to compare the AL, CCT, ACD, CLT, VCD, PD and TZ between the baseline and the values after one year of wearing Ortho-K lenses. Unpaired t-tests were used to compare changes in AL, CCT, ACD, CLT, VCD, PD and TZ between the groups. Ocular parameter changes over time were analyzed using repeated measures analyses of variance (RM-ANOVA). Factors that may affect axial elongation were examined using Pearson correlation analysis. Univariable and multivariable linear regression models were constructed to evaluate the association between each factor and change in AL. Factors with a *P* value less than 0.20 in the univariable analysis were included in the multivariable linear regression analysis with backward variable selection. Standardized regression coefficients from the regression models are presented with 95% confidence intervals (95% CIs). We considered *P* < 0.05 to be statistically significant.

## Results

### Subject demographics

A total of 225 participants were assessed in this retrospective study of which 141 passed the initial screening. Only 75 subjects met the inclusion criteria and were grouped into either the AL elongation group or the AL reduction group (Fig. 1). For the baseline values, such as age, the M/F ratio, SER, AL, and other ocular biometrics, no significant differences (all *P* > 0.05, unpaired t-test and Chi-squared test, Table 1) were found between the groups. Thirty-seven subjects (17 males and 20 females) aged 11.49 ± 1.77 years and 38 subjects (20 males and 18 females) aged 10.92 ± 1.44 years in the AL reduction group and AL elongation group, respectively, attended the one-year follow-up visit. The average SER at the initiation of Ortho-K lens wear was − 3.35 ± 1.25 D and − 2.93 ± 1.14 D, and the baseline ALs were 24.99 ± 0.86 mm and 24.84 ± 0.87 mm in the AL reduction group and AL elongation group, respectively. Detailed subject characteristics are provided in Table 2.

**Table 1:** Demographics and ocular biometrics of the right eyes in each group

Variables	AL reduction group		AL elongation group		*P*
	Mean ± SD	Range	Mean ± SD	Range	
Age (years)	11.49 ± 1.77	8 to 14	10.92 ± 1.44	9 to 14	0.133
Gender (M/F)	17/20		20/18		0.304
SER (D)	− 3.35 ± 1.25	− 5.75 to − 1.25	− 2.93 ± 1.14	− 5.25 to − 1.25	0.128
CRC (mm)	7.81 ± 0.30	7.10 to 8.62	7.80 ± 0.26	7.31 to 8.30	0.774
WTW (mm)	11.99 ± 0.40	11.28 to 12.80	11.98 ± 0.34	11.38 to 12.67	0.864
PD (mm)	3.92 ± 0.60	2.56 to 5.16	3.95 ± 0.55	2.83 to 5.04	0.829
AL (mm)	24.99 ± 0.86	23.43 to 26.82	24.84 ± 0.87	23.40 to 26.70	0.473
CCT (μm)	552.84 ± 22.21	509 to 594	551.21 ± 31.47	478 to 609	0.797
ACD (mm)	3.34 ± 0.19	2.92 to 3.61	3.34 ± 0.20	2.71 to 3.62	0.949
CLT (mm)	3.45 ± 0.16	3.10 to 3.87	3.39 ± 0.16	3.08 to 3.68	0.139
VCD (mm)	17.65 ± 0.85	16.12 to 19.49	17.56 ± 0.89	15.94 to 19.33	0.678

*SER* = spherical equivalent refraction error; *CRC* = corneal radius of curvature; *WTW* = white-to-white corneal diameter; *PD* = pupil diameter; *AL* = axial length; *CCT* = central corneal thickness; *ACD* = anterior chamber depth; *CLT* = crystalline lens thickness; *VCD* = vitreous chamber depth; *M* = male; *F* = female; *D* = diopters; *SD* = standard deviation

*P* values are two-tailed. *P* was calculated using Student’s t-test (unpaired samples). Statistical significance was considered at the 0.05 level

**Table 2:** Summary of results at one-year follow-up

Variables	AL reduction group				AL elongation group				*P*
	Baseline	1 year	*P*	1-year changes	Baseline	1 year	*P*	1-year changes	
AL (mm)	24.99 ± 0.86	24.81 ± 0.86	**< 0.001**	− 0.18 ± 0.06	24.84 ± 0.87	25.13 ± 0.86	**< 0.001**	0.28 ± 0.10	**< 0.001**
CCT (µm)	552.84 ± 22.21	537.11 ± 25.77	**< 0.001**	− 15.73 ± 10.18	551.21 ± 31.47	537.82 ± 34.00	**< 0.001**	− 13.39 ± 8.38	0.281
ACD (mm)	3.34 ± 0.19	3.24 ± 0.20	**< 0.001**	− 0.10 ± 0.08	3.34 ± 0.20	3.32 ± 0.20	0.096	− 0.02 ± 0.07	**< 0.001**
CLT (mm)	3.45 ± 0.16	3.54 ± 0.18	**< 0.001**	0.10 ± 0.10	3.39 ± 0.16	3.42 ± 0.15	**0.002**	0.03 ± 0.06	**0.002**
VCD (mm)	17.65 ± 0.85	17.49 ± 0.84	**< 0.001**	− 0.16 ± 0.07	17.56 ± 0.89	17.84 ± 0.89	**< 0.001**	0.28 ± 0.10	**< 0.001**
PD (mm)	3.92 ± 0.60	4.07 ± 0.61	0.106	0.15 ± 0.49	3.95 ± 0.55	3.99 ± 0.69	0.520	0.02 ± 0.44	0.279
TZ (mm^2^)	–	13.72 ± 3.29	**< 0.001**	–	–	16.44 ± 3.53	**< 0.001**	–	**0.030**

*AL* = axial length; *CCT* = central corneal thickness; *ACD* = anterior chamber depth; *CLT* = crystalline lens thickness; *VCD* = vitreous chamber depth; *PD* = pupil diameter; *TZ* = treatment zone

The changes at one year were calculated by subtracting the baseline from the value after one year of wearing Ortho-K lenses

*P* was calculated using Student’s t test (paired samples); *P* was calculated using Student’s t-test (unpaired samples)

Statistical significance was considered at the 0.05 level. Bold text indicates a significant difference (*P* value < 0.05)

### Changes in axial length

**Fig. 3:**
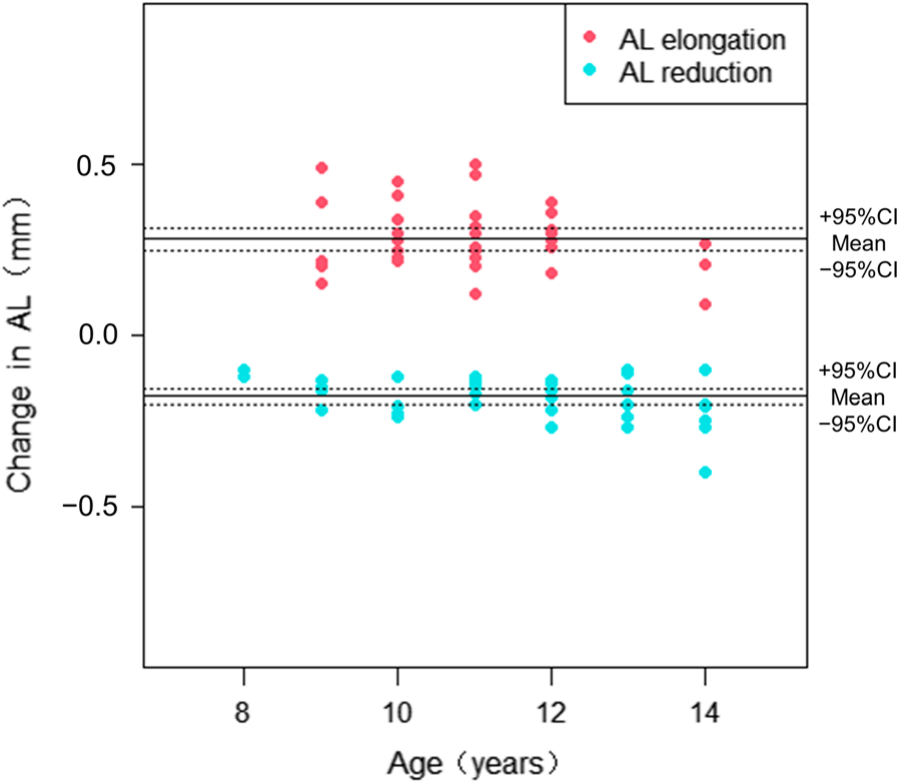
Scatter plots showing differences in the ΔAL and age between the two groups. AL, axial length; Change in AL (ΔAL), AL at the one-year visit − AL at baseline; 95% CI, 95% confidence interval

Over the one-year follow-up, the mean AL reduction was − 0.18 ± 0.06 mm in the AL reduction group, and 16.22% of subjects had a reduction of more than 0.25 mm. The mean axial elongation was 0.28 ± 0.10 mm in the AL elongation group, and 42.11% of subjects had an increase of more than 0.30 mm (Fig. 3). Baseline age was negatively correlated with AL reduction (r = − 0.379, *P* = 0.021). None of the baseline myopic refractive error or other ocular biometric factors had a significant impact on AL reduction (all *P* > 0.05). The AL changes were significantly different between groups (*P* < 0.001, Table 2).

### Differences in ocular parameter characteristics between the two groups

**Fig. 4:**
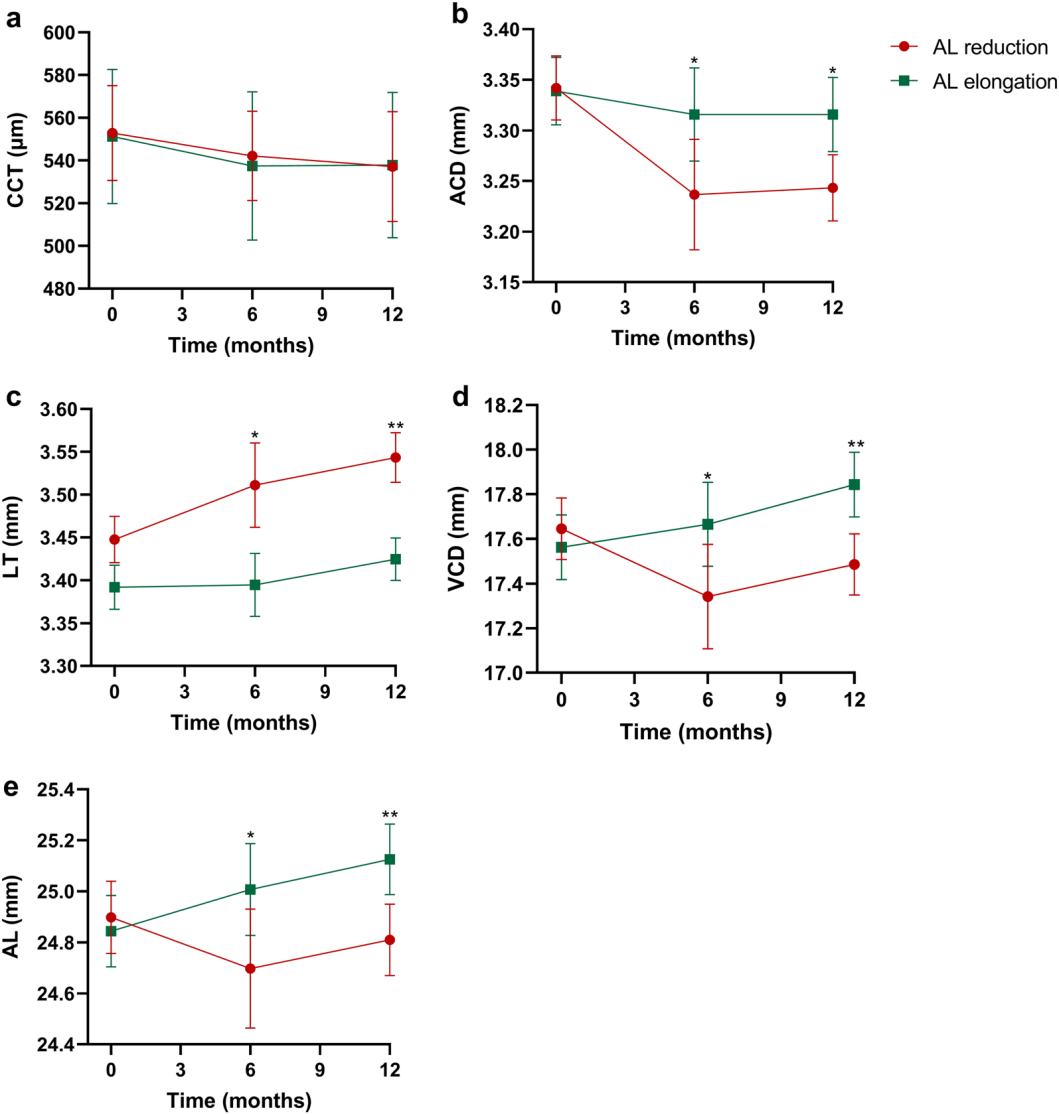
The trend of ocular parameters over one year of Ortho-K treatment in the AL reduction and AL elongation groups. **a** Central corneal thickness (CCT); **b** Anterior chamber depth (ACD); **c** Crystalline lens thickness (CLT); **d** Vitreous chamber depth (VCD); **e** Axial length (AL). Data are expressed as the means and standard error of the mean. Unpaired t-test; *P < 0.05, **P < 0.01 indicate significant differences between the different groups

The trend of ocular parameters over time in the two groups is shown in Fig. 4. After one year of Ortho-K lens wear, the main effect of time on CCT was significant (F = 37.43, *P* < 0.01), whereas the interaction effect of time and groups on CCT was not significant. There was no significant difference between the two groups over the one-year period (*P* = 0.281, Fig. 3a). The interaction effect of time and groups for ACD was significant (time × groups, F = 8.22, *P* < 0.01), and the main effect of time on ACD was significant (time, F = 15.98, *P* < 0.001, Fig. 3b). Similar results were found in CLT (time × groups, F = 5.96, *P* < 0.01; time, F = 13.44, *P* < 001, Fig. 3c). The ACD in the two groups decreased while CLT thickened over time, and the ACD was significantly reduced in the AL reduction group compared with the AL elongation group over a one-year period (*P* < 0.01), while CLT was significantly increased in the AL reduction group compared with the AL elongation group over a one-year period (*P* < 0.01). The interaction effect of time and groups for VCD was significant (time × groups, F = 16.24, *P* < 0.001), and the main effects of time on VCD were significant (time, F = 3.26, *P* < 0.05). A similar trend was found in AL changes (time × groups, F = 28.02, P < 0.001; time, F = 7.15, *P* < 0.05). The VCD and AL were significantly reduced in the AL reduction group but significantly increased in the AL elongation group over a one-year period (*P* < 0.01, Fig. 3d and e).

### Factors associated with reduction in AL after one year of Ortho-K treatment

Associations of AL change with candidate factors are shown in Table 3. In the univariable linear regression model, AL change was significantly associated with baseline age (*P* = 0.031) and TZ (*P* = 0.008) after one year of Ortho-K lens wear. In the multivariable linear regression model, AL change was significantly associated with baseline age (*P* = 0.009) and TZ (*P* = 0.003) after adjustment for age and sex. The standardized regression coefficient in the multivariable regression model was 0.024 (95% CI: 0.009 to 0.040), suggesting that greater AL reduction was associated with older baseline age and a smaller TZ (Fig. 2).

**Table 3:** Univariable and multivariable analyses of the associations between all potential factors and changes in AL

Parameters	Univariable analysis		Multivariable analysis^a^	
	B^b^ (95% CI)	*P*	B (95% CI)	*P*
Age (years)	− 0.038 (− 0.072 to − 0.004)	**0.031**	− 0.047 (− 0.087 to − 0.006)	**0.024**
Gender (M/F)	0.062 (− 0.051 to 0.176)	0.277	0.113 (− 0.032 to 0.257)	0.124
SER (D)	0.033 (− 0.014 to 0.080)	0.162	− 0.04 (− 0.140 to 0.060)	0.276
CRC (mm)	− 0.035 (− 0.240 to 0.170)	0.737	0.579 (− 0.246 to 1.405)	0.164
HVID (mm)	− 0.053 (− 0.210 to 0.104)	0.504	− 0.067 (− 0.341 to 0.207)	0.626
PD (mm)	− 0.021 (− 0.131 to 0.088)	0.697	0.089 (− 0.032 to 0.211)	0.147
AL (mm)	− 0.033 (− 1.00 to 0.033)	0.321	− 0.195 (− 0.469 to 0.078)	0.157
CCT (μm)	− 0.000414 (− 0.003 to 0.002)	0.698	< 0.001 (− 0.002 to 0.003)	0.891
ACD (mm)	− 0.081 (− 0.371 to 0.208)	0.577	0.022 (− 0.549 to 0.592)	0.939
LT (mm)	− 0.199 (− 0.549 to 0.152)	0.263	− 0.276 (− 0.721 to 0.169)	0.218
VCD (mm)	− 0.021 (− 0.088 to 0.045)	0.527	–	–
TZ (mm^2^)	0.023 (0.006 to 0.039)	**0.008**	0.03 (0.012 to 0.049)	**0.002**
Final model	R^2^ = 0.303	*P* = 0.078		

*SER* = spherical equivalent refraction error; *CRC* = corneal radius of curvature; *HVID* = horizontal visible iris diameter; *PD* = pupil diameter; *AL* = axial length; *CCT* = central corneal thickness; *ACD* = anterior chamber depth; *CLT* = crystalline lens thickness; *VCD* = vitreous chamber depth; *TZ* = treatment zone; *M* = male; *F* = female; *D* = diopters. Bold values indicate statistical significance, *P* < 0.05

^a^Adjusted for age and sex

^b^Regression coefficient B is the standardized regression coefficient

## Discussion

This study investigated the baseline characteristics of ocular parameters and variables as well as longitudinal changes in ocular parameters and the TZ in myopic Chinese children over a one-year period. This is one of the first studies to evaluate AL reduction in myopic children treated with the Ortho-K lens for one year follow-up. The results of this study showed that older baseline age and smaller TZ wearing Ortho-K were associated with AL reduction. In myopic children wearing Ortho-K lenses with AL reduction, thickened CLT, decreased ACD and thinned VCD were more obvious during the one-year period, and these changes are helpful for analyzing the biological characteristics of AL reduction which provide clues for further study of the specific mechanism of myopia progression to achieve a better myopia control effect.

### Possible reasons for AL reduction

Although many studies have shown that Ortho-K treatment is effective for myopia control, the mechanism by which Ortho-K might control myopia progression or even AL reduction is still controversial. AL change is a crucial index in the evaluation of myopic progression, and AL reduction due to Ortho-K treatment tends to achieve a much better myopia control effect. Some studies have reported AL reduction after Ortho-K lens wear. Zhao et al. [27] revealed that the six-month AL change was 0.04 ± 0.12 mm, and a decrease in AL was observed in 33% of the eyes treated with Ortho-K lenses. Chen et al. [21] reported that the AL in 49% of eyes treated with Ortho-K lenses for three weeks decreased. However, none of these studies extended a longer period of follow-up, and details about changes in AL components have rarely been reported.

More studies have suggested that choroidal thickness may be one of the factors contributing to AL reduction. The longitudinal orthokeratology research in children (LORIC) study by Cho et al. [28] reported AL reduction in study subjects during the first six months of Ortho-K lens treatment, where increased choroidal thickness was the speculative explanation for AL reduction. In Chen et al.’s study, the estimated mean change in the choroidal thickness was 21.8 ± 25.2 µm after three weeks of Ortho-K lens wear. In our study, the one-year AL value was reduced by 0.18 ± 0.06 mm in the AL reduction group, which was greater than 0.04 ± 0.12 mm as reported by Zhao et al. [27] and 0.03 ± 0.13 mm as reported by Cho et al. [11]. Additionally, 16.22% of the subjects had a reduction of AL of more than 0.25 mm after wearing the Ortho-K lenses even though the choroidal thickness was approximately 0.30 mm [29, 30] and the choroidal thickness change was only approximately 20 µm [21]; hence, it is difficult to explain this phenomenon by choroidal thickness change alone. In addition, the aforementioned studies measured short-term AL changes from baseline; therefore, the response may be a short-term choroidal response to Ortho-K lens treatment rather than a true AL change, and AL reduction in these patients could have led to overestimation of the effect of Ortho-K lens treatment on myopia control. To eliminate the influence of a short-term choroidal response on AL change, we studied changes in ocular parameters in subjects with AL reduction over a period of one year to better understand the long-term effects of Ortho-K lens treatment on the anterior and posterior segments and its effect on AL change. We also tried to control the influence of rhythm and factors on the change in choroidal thickness, but the choroid is a tissue that changes all the time and is affected by many other factors (environment, rhythm, visual stimuli, food, etc.). The thickening of choroidal thickness is one of the reasons for the compensation effect of AL reduction, and the definitive mechanism needs to be further studied.

The CCT change was another possible reason for the AL reduction after Ortho-K treatment that has been observed in many studies. Wang et al. observed 249 Ortho-K wearers over an average period of 2.5 years and reported that CCT was not associated with AL elongation during Ortho-K treatment [31]. Some studies reported a decrease in the CCT [20, 32] after Ortho-K lens wear. In concordance with the findings of these studies, we found CCT reduction beyond the baseline value after Ortho-K lens wear. These changes in the anterior segment of the eye partly explain AL reduction after Ortho-K lens treatment. However, in Chen et al.’s study, the perceived AL change was significantly correlated with the CCT change after Ortho-K lens treatment, but the predictive values were low (12%), indicating that there are other potentially important factors affecting AL. Epithelial change could contribute to the variation in AL, but it may not be the primary mechanism explaining these changes. Furthermore, a significant reduction in the VCD was detected in the AL reduction group. The reductions in the CCT (15.73 µm) and VCD (160 µm) were approximately the same as those in the AL reduction (180 µm), suggesting that the AL reduction was mainly due to the shortening of the VCD. We hypothesize that the reduction in AL is partly due to choroidal thickening pushing the retina forward, resulting in a shortened VCD, but there may be other factors involved. Another remarkable finding of this study was the similarity between the ACD reduction (100 µm) and the CLT increase (100 µm). This indicates that the change in the ACD may be caused by the thickening of the CLT.

As mentioned previously, we found that in children with AL reduction, their crystalline lens is thickened, corresponding to a shortened ACD. Some previous studies have reported relationships between ocular components and relative peripheral refraction, which may trigger AL elongation in myopic participants [33]. They found that a deeper ACD, a deeper vitreous chamber [34–36] and a thinner CLT [37] were associated with hyperopic relative peripheral refraction in myopic children, indicating a tendency for longer eyes and that a shallower ACD, thicker CLT and thinner VCD may decrease hyperopic relative peripheral refraction, slowing axial elongation. A study by Li et al. found that CLT increased during one year of Ortho-K lens treatment but did not recover after one month of lens cessation [19]. Wang et al. also observed that a greater increase in CLT was associated with a smaller increase in AL at the 12-month follow-up during Ortho-K wear in children with myopia [38]. These results may indicate that the continuous wearing of Ortho-K lenses may result in a change in the crystalline refractive power, which may remain stable and nonreversible in the short term, correlating with a smaller increase in AL. Another study found a significant ACD reduction during Ortho-K treatment, and the ACD reduction at the center of the cornea was similar to the significant AL reduction that accompanied it [39]. Consistent with these studies, we also observed a reduced ACD and VCD and an increased CLT in the AL reduction group compared with those in the AL elongation group. However, increased CLT might also be considered a result of AL reduction as more accommodation could be introduced as compensation for overcorrection caused by shortening of AL.

Notably, in this study, 16.22% of subjects had an AL reduction of more than 0.25 mm, which is difficult to explain by changes in corneal thickness and choroidal thickness, as the magnitude of the changes exceeds that for which the cornea and choroid can compensate. Therefore, we speculate that the sclera may have remodeled during the process of AL reduction. Previous studies have confirmed that the choroid, a vascular structure between the retina and the sclera, supplies RPE and the outer retina. In addition, it contains secretory cells, which may be involved in mediating scleral growth. Thus, the choroid has a crucial role in relaying signals derived from the retina to the sclera, further altering the synthesis of scleral extracellular matrix molecules and changing the size of the eye. Therefore, we hypothesized that choroidal thickening after wearing Ortho-K lenses might modulate the sclera and thus further affect AL. The exact mechanism underlying the results observed in this study remains to be explored.

### Factors affecting the Ortho-K control effect

Some studies have reported that the effect of Ortho-K on the control of myopia has great individual variability, which may be affected by age at the initiation of Ortho-K wear [25, 40]. The same conclusion was also reached in this study. In addition, the TZ is also a factor affecting the progression of myopia. As mentioned previously, changes in peripheral defocus and aberration have been hypothesized as explanations for the mechanism of Ortho-K in myopia control [41, 42]. Lau et al. [43] analyzed 103 myopic children treated with Ortho-K lenses over a two-year follow-up and found that ocular higher-order aberrations, particularly spherical aberrations, had a negative correlation with axial elongation. Increasing higher-order aberration may slow axial elongation in Ortho-K treatment. In addition, myopia control of Ortho-K is associated with relative peripheral refraction. According to studies by Carracedo et al. [44] and Guo et al. [25], a smaller TZ tends to cause more myopic relative peripheral refraction and a larger change in higher-order aberrations, achieving an improvement in myopia control. Consistent with their findings, our study found that children in the AL reduction group had a smaller TZ than those in the AL elongation group in the absence of a statistically significant difference in pupil size between groups, and AL reduction was significantly positively correlated with the TZ. This result suggests that a smaller TZ plays a potential role in controlling AL elongation using Ortho-K and provides a new perspective on the personalized design of Ortho-K lenses for myopic children.

### Limitations

Our study had some limitations. First, it would have been ideal to accurately measure SER after Ortho-K treatment to further confirm the degree of myopia progression in this study. In addition, some studies have revealed that AL changes are significantly correlated with choroidal thickness changes after Ortho-K treatment; however, we did not evaluate the change in choroidal thickness after Ortho-K lens wear. Therefore, further studies should assess changes in the anterior and posterior segments to predict AL changes in Ortho-K patients. In addition, the one-year observation period chosen for this study is because most myopic children treated with the Ortho-K lens change their lenses after approximately one year of wear in view of safety concerns. For cases of AL reduction, a longer follow-up period is still needed to observe the effect of AL control. In addition, the relatively small number of children with AL reduction limited the sample size of this study, and larger sample sizes should be undertaken in future studies.

## Conclusion

Baseline age was negatively associated with AL change, and TZ was positively associated with axial length change in myopic children treated with Ortho-K. Thickened CLT, decreased ACD and thinned VCD were observed in AL reduction eyes compared with AL elongation eyes with Ortho-K wearing. The changes in CLT, ACD, and VCD may suggest a better myopia control effect using Ortho-K. Thickened CLT may compensate for overcorrection in AL reduction eyes.

## Data Availability

All data generated or analyzed during this study are included in this published article.
